# Analysis of Intentional Electromagnetic Interference Effects on PWM Command Interpretation in UAV BLDC Motor Controllers

**DOI:** 10.3390/s26123881

**Published:** 2026-06-18

**Authors:** Hyunsu Cho, Euijin Kim, Wonsuk Choi

**Affiliations:** School of Cybersecurity, Korea University, Seoul 02841, Republic of Korea; hscho20@korea.ac.kr (H.C.); goodlina12@korea.ac.kr (E.K.)

**Keywords:** intentional electromagnetic interference, UAV security, electronic speed controller, pulse-width modulation, physical-layer attack, motor stoppage, brushless DC motor

## Abstract

Multirotor unmanned aerial vehicles (UAVs) rely on electronic speed controllers (ESCs) that decode motor commands from pulse-width modulation (PWM) signals, making the flight-controller-to-ESC command path a physical-layer attack surface for intentional electromagnetic interference (IEMI). This paper presents a mechanism-based analysis of IEMI attacks that induce motor stoppage in UAV brushless DC motor controllers. We develop a timing-error model in which a sinusoidal disturbance on the PWM line shifts the detected edge instants and drives the decoded pulse width into stop-equivalent regimes, and we show that the disturbance reaching the ESC’s thresholding node is shaped by a frequency-selective cascade of the PWM cable’s coupling response and the ESC’s input-path transfer function. We experimentally characterize this model on five commercial ESCs through conducted and radiated injection. The measured thresholds differ by more than an order of magnitude across ESCs and are reordered between frequency bands and injection modes; comparing conducted and radiated results allows us to attribute these differences primarily to the cable coupling response and reveals cases where it either hides or amplifies an ESC’s susceptibility. The susceptible frequency also shifts with PWM cable length in qualitative agreement with transmission-line resonance, confirming that observed radiated susceptibility reflects the joint design of ESC and cable rather than a single intrinsic property. The cable lengths examined here (45–125 cm) are longer than those of compact multirotors and were chosen to place resonances within our antenna’s band; we discuss the implications of this choice and identify shorter, deployment-realistic cables as a priority for future work.

## 1. Introduction

Unmanned aerial vehicles (UAVs), commonly referred to as drones, are being adopted in an increasingly broad range of applications, including logistics, inspection, surveillance, emergency response, and infrastructure monitoring. As UAV deployment expands from hobbyist platforms to safety- and mission-critical operations, the security and reliability of their low-level control stack become increasingly important. In particular, multirotor UAVs rely on a tightly coupled sensing–control–actuation loop, in which even small deviations in rotor thrust can rapidly translate into attitude instability or loss of control. Within this loop, the electronic speed controller (ESC) serves as a safety-critical actuator interface: it translates commands from the flight controller (FC) into three-phase switching actions that regulate brushless DC (BLDC) motor speed. Although newer digital ESC protocols have gained traction, pulse-width modulation (PWM) remains widely used in commodity and legacy-compatible UAV platforms, making the integrity of the flight-controller-to-ESC command path a practical security concern [1,2].

A growing body of research has shown that intentional electromagnetic interference (IEMI) can violate the integrity of cyber-physical systems by exploiting wiring, front-end circuitry, and threshold-based digitization. Prior work on out-of-band and electromagnetic (EM) signal injection has established that injected high-frequency interference can be transformed into meaningful corruption at lower layers of system operation through coupling paths, nonlinear circuit behavior, and digitization stages [3,4,5]. These studies collectively suggest that physical-layer interference is not merely a reliability issue but also a security threat capable of manipulating sensing, communication, and control without compromising software.

Such threats have already been demonstrated in UAV-related contexts. In the drone domain, Jang et al. showed that electromagnetic signal injection can paralyze UAVs by corrupting sensor communication channels, highlighting the existence of susceptible frequency regions that enable reliable disruption [6]. In the actuator domain, Dayanikli et al. showed that IEMI can jam or alter PWM-controlled actuators, thereby establishing PWM as a direct physical-layer attack surface [1]. However, that line of work mainly focused on servo-like PWM actuators, where the actuation target is primarily positional control. By contrast, the propulsion system of a multirotor UAV is governed by BLDC-ESCs whose interpreted PWM command directly determines motor speed and, ultimately, thrust. Errors in the decoded command, therefore, translate directly into loss of propulsion, coupling into the UAV flight-control loop with potentially catastrophic consequences.

Despite these advances, the propulsion command path of commodity BLDC-ESCs remains insufficiently understood from a physical-layer security perspective. Although prior work has shown that electromagnetic interference (EMI) injection on a PWM line can, in principle, corrupt its interpretation, attempting such an attack in practice produces markedly inconsistent results: the same injection frequency and amplitude that halt one ESC may leave a different ESC—or even the same ESC in a different installation—entirely unaffected. Existing studies have neither systematically analyzed how IEMI perturbs the threshold-based edge-timing pipeline used in PWM decoding inside ESCs, nor quantitatively related ESC- and deployment-specific characteristics—such as the input-path filter and the length of the PWM cable—to these observed differences. As a consequence, the combinations of injection frequency and amplitude under which an attacker can reliably induce motor stoppage, and the reasons why the same attack succeeds against some ESCs but not others, remain poorly characterized across real ESC implementations.

**Figure 1:**
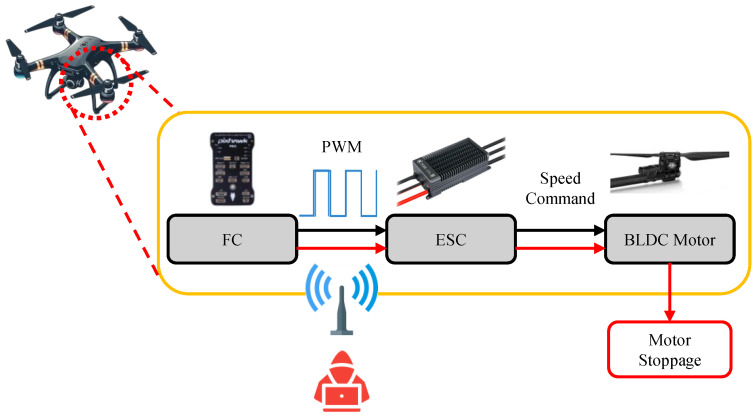
Overview of electromagnetic interference (EMI)-induced corruption of the flight controller (FC)-to-electronic speed controller (ESC) pulse-width modulation (PWM) command path and its impact on brushless DC (BLDC) motor behavior.

Figure 1 provides an overview of the problem addressed in this paper. A sinusoidal EMI signal coupled onto the FC-to-ESC PWM line perturbs the threshold-crossing times used for PWM decoding, causing the ESC to reconstruct an unintended pulse width even when the FC output remains unchanged. When the corruption is sufficient, the ESC’s decoded command enters one of several stop-equivalent regimes—invalid-signal failsafe, minimum-throttle decoding, or edge-capture timeout—and the motor ceases to produce thrust. Beyond this high-level attack chain, the figure also emphasizes the central analytical focus of this paper: explaining how ESC-specific input-path characteristics and the PWM cable’s coupling response jointly determine the injection frequencies and amplitudes at which this attack succeeds.

This paper addresses these gaps by providing a mechanism-first analysis of IEMI attacks against BLDC-ESCs used in UAV propulsion systems. We develop a timing-error model that describes how a sinusoidal disturbance superimposed on the PWM line shifts the detected rising and falling edges, and we formalize the motor-stoppage condition as a requirement on the cumulative pulse-width error. We then show that the disturbance amplitude actually reaching the ESC’s thresholding node is shaped by the cascade of the PWM cable’s coupling response and the ESC’s input-path transfer function, both of which are frequency-selective. The model predicts that each ESC exhibits a characteristic curve of minimum injection amplitude as a function of injection frequency, and that the shape of this curve directly reflects the ESC’s design attributes and the cable configuration in which it is deployed. We experimentally characterize this prediction through conducted and radiated injection experiments on five commercial ESCs, measuring how the minimum stoppage amplitude varies with frequency, ESC model, and cable length.

### Contributions

This paper makes three main contributions.

- We develop a timing-error model that explains how a sinusoidal EMI signal injected onto the PWM line shifts the ESC’s threshold-crossing times and drives the decoded pulse width into stop-equivalent regimes, inducing motor stoppage.
- We show that the effective disturbance at the ESC’s thresholding node is determined by a cascade of the PWM cable’s coupling response and the ESC’s input-path transfer function, yielding a predicted curve of minimum injection amplitude as a function of frequency.
- We experimentally characterize this prediction on five commercial ESCs through conducted and radiated injection, demonstrating that each ESC exhibits its own frequency-dependent attack threshold curve whose relative ordering changes between frequency bands and between conducted and radiated configurations. We further show that comparing conducted and radiated thresholds attributes the difference primarily to the cable coupling response and reveals cases where it substantially hides or amplifies an ESC’s susceptibility, and that the susceptible frequency shifts with PWM cable length in qualitative agreement with transmission-line resonance.

## 2. Related Work

This section reviews prior work across three areas that converge on the problem addressed in this paper—electromagnetic signal injection attacks on cyber-physical systems, physical-layer threats in UAV platforms, and EMI effects on actuator and motor-drive systems—and positions our contribution relative to each. In brief, while prior work has established IEMI as a physical-layer threat across sensors, communication buses, and servo-like actuators, the propulsion command path of UAV BLDC-ESCs has not been analyzed at the mechanism level; the present paper develops a timing-error model for this path and characterizes how ESC- and cable-specific factors shape its susceptibility, as detailed below.

### 2.1. Electromagnetic Signal Injection Attacks on Cyber-Physical Systems

A substantial body of work has established that intentional electromagnetic interference can compromise the integrity of cyber-physical systems without requiring software-level access. Kune et al. demonstrated that low-power EMI can inject ghost signals into analog sensor front-ends by exploiting the nonlinear rectification behavior of input circuitry, showing that medical devices such as cardiac implants are vulnerable to spoofed sensor readings [5]. Giechaskiel et al. provided a taxonomy and framework for out-of-band signal injection attacks across sensing modalities, identifying common patterns in how physical-layer interference is transduced into digital-domain corruption through coupling paths, nonlinear circuit elements, and threshold-based digitization [3,4]. Selvaraj et al. showed that IEMI can exploit the nonlinearities of electrostatic discharge (ESD) protection circuits to precisely toggle voltage levels on digital lines, enabling targeted manipulation of servo motor angles [7]. Yan et al. formalized the analog sensor attack surface and proposed a minimalist framework for reasoning about the security of sensor systems under physical-layer threats [8].

More recent work has extended electromagnetic injection to digital communication buses and camera systems. Köhler et al. demonstrated signal injection attacks against charge-coupled device (CCD) image sensors, showing that crafted EMI can introduce adversarial perturbations into captured images [9]. Jiang et al. showed that IEMI can corrupt image data during cable transmission between camera sensors and processors, affecting object detection in safety-critical applications [10]. Wang et al. demonstrated that electromagnetic injection can target differential signaling protocols such as the Controller Area Network (CAN) bus, which are widely used in automotive and aviation systems [11]. These studies collectively establish that physical-layer electromagnetic injection is a broadly applicable attack vector, but the specific mechanisms and failure modes are highly dependent on the target interface, circuit topology, and digitization process.

### 2.2. Physical-Layer Threats to UAV Platforms

UAVs present a particularly rich attack surface due to their reliance on multiple sensing, communication, and actuation subsystems. Prior work has targeted various layers of this stack. Global Positioning System (GPS) spoofing attacks have been shown to redirect UAV flight paths by injecting counterfeit satellite signals [12,13]. Acoustic injection attacks have exploited the mechanical resonance of microelectromechanical systems (MEMS) gyroscopes and accelerometers to corrupt inertial measurement data, although such attacks are sensor-specific and can be mitigated by sensor diversity or acoustic shielding [14,15,16].

Most directly relevant to this paper, Jang et al. demonstrated that EMI signal injection can paralyze multirotor drones by corrupting the Inter-Integrated Circuit (I2C) communication channel between the inertial measurement unit (IMU) sensor and the FC [6]. Their work showed that each control unit board has a distinct susceptible frequency at which EMI most effectively distorts the transmitted sensor data, and that the attack is effective regardless of the specific IMU sensor model. This result established that digital communication buses within UAVs are vulnerable to electromagnetic injection at specific frequencies. However, their attack targets the sensing path (IMU-to-FC communication), whereas the present paper targets the actuation path (FC-to-ESC PWM command). The distinction is significant: corrupting sensor data affects the FC’s state estimation, while corrupting the PWM command directly alters the thrust produced by individual motors, bypassing the FC’s decision-making entirely.

Esteves et al. conducted early experimental studies on the effects of IEMI on civilian UAVs, demonstrating that strong electromagnetic fields can induce various malfunctions [17]. Kim et al. characterized the electromagnetic signature of quadcopter drones and analyzed coupling mechanisms that determine how external fields interact with UAV electronics [18]. A recent survey by Lee et al. provides a broad overview of IEMI threats to UAV sensor modules, categorizing attacks by power level, required information, and target frequency [19]. While these studies establish the general vulnerability of UAV platforms to electromagnetic interference, they do not provide mechanism-level analysis of how specific subsystems—particularly the propulsion command path—fail under targeted injection.

### 2.3. EMI Effects on Actuators and Motor-Drive Systems

The closest prior work to our contribution is the line of research by Dayanikli et al. on IEMI attacks against PWM-controlled actuators [1,20]. They demonstrated that electromagnetic interference can jam or alter PWM signals used to control servo motors and power converters, establishing PWM as a direct physical-layer attack surface. Their work showed that IEMI can either suppress the PWM command (causing actuator loss) or shift the decoded pulse width (causing unintended actuation), and proposed hardware-level countermeasures, including fiber-optic signal transmission and printed circuit board (PCB)-level filtering. Asif et al. further explored the feasibility of confidential PWM communication to protect actuator commands from electromagnetic eavesdropping and injection [2].

However, several gaps remain between this prior work and the problem addressed in this paper. First, Dayanikli et al. focused primarily on servo-like actuators in which the PWM command controls angular position, with the attack target being a specific decoded angle. In contrast, BLDC-ESCs used in UAV propulsion interpret PWM commands as speed references and enforce additional validity checks—failsafe on out-of-range widths, minimum-throttle decoding, and edge-capture timeouts—that open several distinct paths to motor stoppage. A propulsion-focused attack model, therefore, requires reasoning about these stop-equivalent regimes together, rather than about a single decoded value. Second, prior work has largely reported attack feasibility at individual frequencies or in qualitative terms, without developing a quantitative model that predicts how the minimum injection amplitude varies with frequency across a target device. The timing-error model developed in this paper, combined with the cascade of cable and input-path transfer functions, provides such a prediction and connects it directly to measurable attack thresholds. Third, existing studies have not systematically examined how ESC-specific front-end characteristics—most notably the input resistor–capacitor (RC) filter—and deployment-specific factors such as the PWM cable length jointly shape the susceptible frequency band. The cross-device and cable-length experiments presented in this paper provide this missing link between ESC design attributes, physical installation, and observable attack conditions.

In the broader motor-drive literature, electromagnetic compatibility (EMC) studies have extensively characterized EMI effects on variable-speed drives and BLDC motor controllers from a reliability and compliance perspective [21,22]. These studies typically focus on emissions from the drive system rather than susceptibility to external injection, and they address unintentional interference rather than adversarial exploitation. Our work reframes the EMC problem as a security question: rather than asking how to ensure regulatory compliance, we ask what an adversary can achieve by deliberately injecting interference at frequencies and amplitudes chosen to maximize the impact on propulsion behavior.

## 3. Background

### 3.1. BLDC Motor Operation and PWM Command Encoding

Multirotor UAVs generate thrust using BLDC motors, in which phase currents are switched electronically rather than through mechanical brushes. In a typical PWM-based propulsion stack, the FC specifies the desired motor speed by transmitting a PWM signal to the ESC. The command is encoded in the pulse width, i.e., the duration of the high state within each PWM period. In many conventional PWM interfaces, the pulse width ranges approximately from 1 ms to 2 ms, corresponding to minimum and maximum throttle, respectively [1], with pulse-width values outside this interval treated as invalid signals that trigger the ESC’s failsafe response. The ESC measures this width, maps it to an internal throttle or speed reference, and drives the motor by supplying the corresponding three-phase current sequence. Since the decoded pulse width directly determines the motor-speed command, the integrity of the PWM signal is a prerequisite for stable flight, and any corruption that drives the decoded width outside the valid range or prevents its successful measurement causes the ESC to halt motor drive.

### 3.2. EM Coupling onto the PWM Command Path

When an external electromagnetic source is placed near the FC-to-ESC wiring, its time-varying field couples into the PWM command path through the signal-return loop formed by the PWM conductor and its ground return. This coupling is not uniform across frequency: at low frequencies, it is dominated by inductive pickup, whereas at higher frequencies, skin and proximity effects, parasitic capacitance on wiring and PCB traces, and resonances of the signal-return loop reshape the response. The net coupling efficiency from the external source to the ESC input is therefore a bandpass-like function of frequency, with one or more peaks rather than a monotonic rise. Only at frequencies near these peaks does a given source power produce sufficient voltage at the ESC input to disturb the PWM interpretation.

One concrete contributor to these peaks is the electrical length of the FC-to-ESC cable, which, together with its ground return, forms a transmission-line structure [23]. When the cable length is comparable to a quarter wavelength of the injected signal, standing-wave resonance amplifies the voltage at the ESC input relative to the injection point. In multirotor UAVs, FC-to-ESC cable lengths of roughly 5–30 cm place the corresponding quarter-wave resonances in the sub-GHz band, and the resonant frequency shifts with cable length. This implies that the susceptible frequency band of a given ESC is not solely a property of its hardware but also of the physical installation, a dependence we examine experimentally in Section 6.

**Figure 2:**
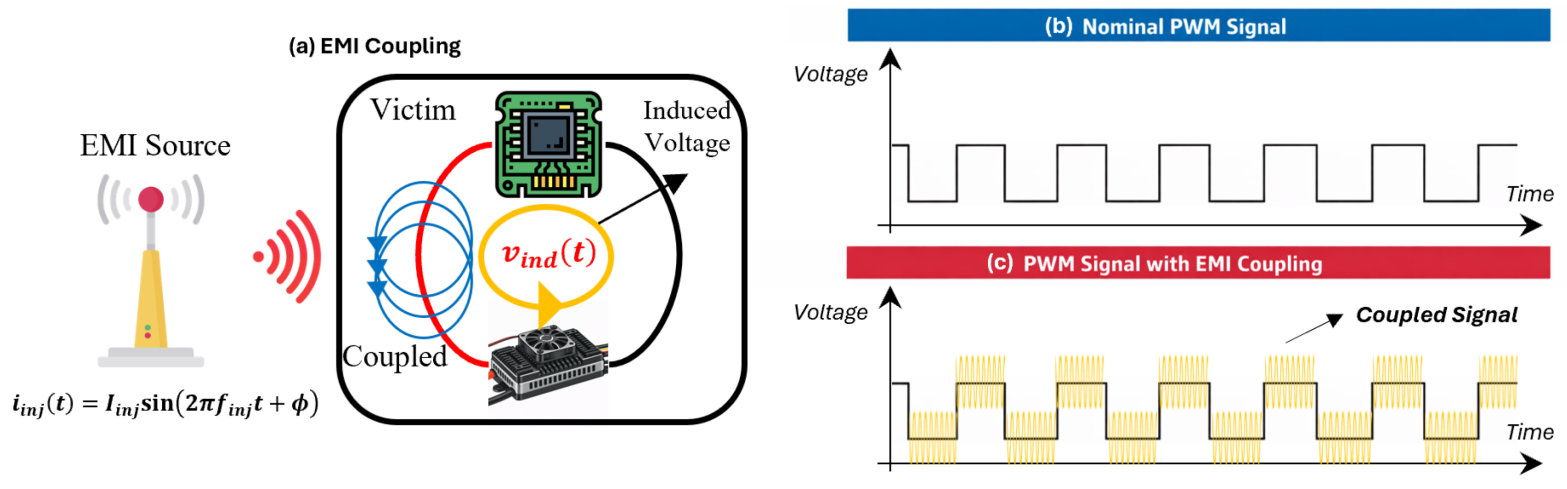
Illustration of EMI coupling and its effect on the PWM command waveform.

Because the FC-to-ESC PWM interface is a simple single-ended signal path and typically lacks physical-layer integrity protection, any disturbance coupled onto the command line is indistinguishable from a legitimate PWM signal at the ESC input. Figure 2a illustrates this coupling process. The resulting voltage perturbation at the ESC input depends on the coupling geometry, loop area, cable length, parasitic circuit elements, and injection frequency.

### 3.3. PWM Interpretation

The ESC recovers the motor-speed command by measuring the pulse width of the incoming PWM signal. Although PWM is logically a digital interface, the waveform at the ESC input is an analog voltage. The microcontroller unit (MCU) digitizes this signal through threshold-based interpretation: a rising edge is recognized when the voltage exceeds the input-high threshold $V_{\mathrm{IH}}$, and a falling edge is recognized when it drops below the input-low threshold $V_{\mathrm{IL}}$. The decoded pulse width is the time interval between these two events:

(1) $$w=t_{\mathrm{fall}}-t_{\mathrm{rise}}.$$

This process makes the ESC fundamentally sensitive to threshold-crossing timing: any perturbation that shifts the detected edge times will alter the decoded pulse width. Figure 2b shows a nominal PWM signal with well-defined edges, whereas Figure 2c shows the same command under EMI coupling, where the superimposed disturbance can shift the threshold-crossing instants even though the FC output remains unchanged.

## 4. Attack Model

We consider a black-box attacker whose objective is to induce motor stoppage in a multirotor UAV through electromagnetic interference on the FC-to-ESC PWM command path, without requiring physical modification of the operational target, firmware compromise, or access to debug interfaces. Motor stoppage, as defined in this paper, is the condition in which the motor’s rotational speed collapses to zero as a result of EMI-induced corruption of the ESC’s PWM interpretation pipeline. We intentionally define the attack outcome at the level of observable motor behavior rather than at the level of internal ESC state, because the ESC’s response to a corrupted PWM signal may involve several indistinguishable failure paths—invalid-signal failsafe, minimum-throttle decoding, or edge-capture timeout—all of which produce the same externally observed result. The attacker can control the injection frequency $f_{\mathrm{inj}}$ and amplitude $A_{\mathrm{inj}}$ of a narrowband sinusoidal source, and does not know the ESC’s internal circuit implementation, timer configuration, or filtering structure.

We assume the attack proceeds in two phases. In an offline characterization phase, the attacker has temporary physical access to a representative instance of the target ESC model and can perform conducted injection onto the PWM line to identify susceptible frequencies. In the subsequent deployment phase, the attacker has no physical access and uses radiated injection at the previously identified frequencies to target operational UAVs equipped with the same ESC model. This two-phase model reflects a realistic adversary who first profiles commercially available hardware in a controlled setting and then mounts a non-invasive attack in the field.

The target of the attack is the PWM-based command interpretation inside the ESC. The attack seeks to induce a pulse-width interpretation error

(2) $$e_{k}={\hat{w}}_{k}-w_{k},$$

where $w_{k}$ is the nominal pulse width of the *k*-th PWM command and ${\hat{w}}_{k}$ is the pulse width decoded by the ESC under interference. For each ESC, there is a minimum sustained magnitude of this error, denoted $e_{\mathrm{stop}}$, beyond which one of the ESC’s stop-equivalent failure paths is triggered, and motor stoppage occurs. The attacker’s operational goal is therefore to drive $|e_{k}|\geq e_{\mathrm{stop}}$ using the minimum possible injection amplitude, which, as Section 5 shows, is a function of the chosen injection frequency.

### Attack Strategy: Path Isolation via Conducted Injection

EMI may, in principle, couple into a UAV through multiple physical paths, including the PWM command line, the power/ground network, the motor-phase wiring, and the sensor-FC communication buses. To establish that the PWM interpretation path is the actual origin of the motor anomalies we study, we adopt a two-stage strategy that first isolates this path experimentally and then verifies that the same vulnerability is exploitable under realistic radiated conditions.

In the first stage, we perform conducted injection—also referred to as wired injection—by directly coupling the attack signal onto the PWM signal cable between the FC and the ESC. Because the injected energy is confined to the PWM conductor and its ground return, this configuration does not radiate electromagnetic fields into the surrounding hardware: other subsystems—power wiring, motor phases, IMU-FC buses—are not appreciably exposed to the injected signal. Consequently, any motor anomaly observed under conducted injection must originate from corruption of the PWM interpretation path rather than from interference with other components. Sweeping the injection frequency and recording the minimum injection amplitude required to induce motor stoppage yields a per-ESC susceptibility curve, and the frequency at which this minimum amplitude is smallest is identified as the resonant frequency of the PWM command path for that device.

In the second stage, we perform radiated injection—also referred to as wireless injection—at the resonant frequencies identified in the first stage, using an antenna at the standoff and polarization specified in Section 6.1. The role of this stage is twofold. First, it verifies that the same vulnerability remains exploitable under a genuinely non-invasive, over-the-air attack matching the deployment-phase threat model. Second, and more importantly for path attribution, agreement between the effective frequency bands under conducted and radiated injection provides evidence that the PWM command path is also the primary entry point under radiated attack; if radiated injection at these frequencies succeeded while alternative coupling paths were the true cause, such agreement would not be expected.

This two-stage strategy directly supports the attacker model defined above. The conducted stage corresponds to the offline characterization phase in which the attacker profiles a representative target device in a controlled setting, while the radiated stage corresponds to the deployment phase in which the attacker acts non-invasively on an operational UAV. It also establishes, through path isolation rather than mere correlation, that the PWM interpretation path is the security-critical component studied in the remainder of this paper.

## 5. Attack Mechanism and Timing Error Model

This section develops the mechanism by which a sinusoidal EMI signal, injected near the FC-to-ESC command path, corrupts the ESC’s interpretation of the PWM signal and ultimately forces the motor into a stopped state. The argument proceeds in three steps. First, we describe how the superposition of a sinusoidal disturbance on the PWM line shifts the threshold-crossing instants used for pulse-width decoding, and how such timing corruption can lead to motor stoppage through several possible failure paths. Second, we show that the ESC’s analog front-end and the physical PWM cable jointly act as a frequency-selective filter, so that the disturbance amplitude effectively reaching the thresholding node depends strongly on the injection frequency. Third, we combine these two effects to argue that, for any given ESC, there exists a specific pairing of injection frequency and minimum injection amplitude that reliably induces motor stoppage.

### 5.1. EMI-Induced Timing Corruption and Motor Stoppage

The ESC decodes the motor-speed command by measuring the pulse width of the PWM signal, i.e., the duration over which the waveform exceeds the logic-high threshold $V_{\mathrm{IH}}$. Under normal operation, the pulse width $w_{k}$ for the *k*-th PWM period, typically bounded between approximately 1 ms and 2 ms, maps directly to a throttle reference.

When a sinusoidal EMI signal is injected onto the PWM line, the waveform observed at the ESC’s thresholding node becomes the superposition

(3) $$x_{k}(t)=u_{k}(t)+n_{k}(t),$$

where $u_{k}(t)$ is the nominal PWM signal and $n_{k}(t)$ is the effective disturbance at the thresholding node. Because the ESC’s digitization is purely threshold-based, the decoded pulse width is determined not by the intended edges of $u_{k}(t)$ but by the actual crossings of $x_{k}(t)$ through the logic thresholds—a mechanism that prior work has exploited to manipulate digital signals on embedded systems [1,7]. A first-order expansion around a nominal crossing instant $t_{0}$ yields the timing shift

(4) $$\Delta t\approx -\frac{n_{k}(t_{0})}{u_{k}^{\prime }(t_{0})},$$

which is a first-order (small-signal) approximation whose regime of validity rests on three assumptions: (i) the effective disturbance amplitude ${\left|n_{k}\right|}_{\mathrm{eff}}$ at the crossing instant is small relative to the logic swing $V_{\mathrm{sw}}$ (nominally 3.3–5 V), so that the local slope $u_{k}^{\prime }(t_{0})$ can be treated as constant over the displacement Δt; (ii) the disturbance does not introduce additional threshold crossings within a single edge transition, i.e., a single, monotone crossing is preserved; and (iii) the edge transition itself is approximately linear over the neighborhood of $t_{0}$. The magnitude of the timing shift grows with the disturbance amplitude at the crossing instant and shrinks with the local edge slope of the filtered PWM waveform.

We note that the assumption ${\left|n_{k}\right|}_{\mathrm{eff}}\ll V_{\mathrm{sw}}$ is well satisfied for the most susceptible devices but weakens for the most robust ones. The lowest stoppage threshold we observe, 180 mV (EC-X3), is only 3.6–5.5% of a 3.3–5 V swing and lies firmly in the linear regime; in contrast, the highest thresholds (e.g., 2.6–2.7 V for Edge 75) approach the same order of magnitude as the swing, where the linearization in (4) degrades and the nonlinear mechanisms discussed below—Schmitt-trigger hysteresis, clipping, and multiple edge crossings—increasingly govern the stop transition. The model is therefore best read as a mechanism that explains the location of the susceptible frequency and the shape of the amplitude-versus-frequency curve, rather than as a quantitatively exact predictor of the absolute threshold for every device. Applying this shift to both edges of the *k*-th pulse, the resulting pulse-width error is the difference between the falling-edge and rising-edge shifts:

(5) $$e_{k}=\Delta t_{\mathrm{fall},k}-\Delta t_{\mathrm{rise},k}.$$

When $\left|e_{k}\right|$ is small, the decoded pulse width ${\hat{w}}_{k}=w_{k}+e_{k}$ remains close to $w_{k}$ and the motor continues to rotate near its nominal speed. As the disturbance grows, several failure paths can cause the ESC to enter a stopped or disarmed state. The decoded pulse width may fall outside the ESC’s valid throttle range, in which case the ESC treats the signal as invalid and invokes its failsafe routine. It may be pushed toward the minimum-throttle value of the ESC’s valid range, which is interpreted as an idle or stop command. Alternatively, severe disturbance near the PWM edges can produce ringing or multiple threshold crossings that prevent successful edge capture, causing the ESC’s timing logic to time out and treat the signal as lost. Common ESC firmware responds to any of these conditions by halting the motor drive. From a black-box attacker’s perspective—and from the perspective of the experiments in this paper, which observe motor behavior rather than internal ESC state—these paths are indistinguishable, and we characterize the attack outcome uniformly as motor stoppage: the condition in which the motor’s rotational speed collapses to zero as a consequence of EMI-induced corruption of the PWM interpretation pipeline.

What the three paths share is a common prerequisite: the effective disturbance at the thresholding node must be large enough that the cumulative timing error $\left|e_{k}\right|$, sustained over multiple PWM periods, drives the ESC’s decoded command into one of these stop-equivalent regimes. This prerequisite motivates the next question: given a sinusoidal injection at the attacker’s source, what determines the disturbance amplitude actually reaching the thresholding node?

### 5.2. Frequency-Selective Attenuation of the Injected Signal

A naive attacker model would assume that the disturbance $n_{k}(t)$ at the thresholding node equals the injected signal in amplitude. In practice, this is not the case: the injected signal traverses the PWM cable and passes through the ESC’s analog front-end before reaching the thresholding node, and both stages shape its amplitude as a function of frequency.

The ESC’s input path typically includes a simple RC low-pass filter on the PWM input line, consisting of a series resistor and a shunt capacitor to ground, together with any parasitic capacitance and protection elements present on the PCB. This stage has a cutoff frequency $f_{c}=1/(2\pi RC)$, above which the injected disturbance is attenuated at approximately 20 dB per decade. Denoting the aggregate input-path transfer function as H(f), a sinusoidal injection of amplitude $A_{\mathrm{inj}}$ at frequency $f_{\mathrm{inj}}$ produces an effective disturbance amplitude at the thresholding node of

(6) $${\left|n_{k}\right|}_{\mathrm{eff}}\approx \left|H(f_{\mathrm{inj}})\right|\cdot A_{\mathrm{inj}}.$$

Two consequences follow. First, ESCs with aggressive input filtering (low $f_{c}$) require substantially higher injection amplitude for attack frequencies above $f_{c}$, and may be effectively immune at sufficiently high frequencies. Second, ESCs with minimal input filtering pass the disturbance to the thresholding node with little attenuation, exposing a wide susceptible bandwidth.

The PWM cable itself also contributes to the frequency response, and in a non-trivial way. The cable, together with its ground return and the chassis/connector elements at either end, forms a loaded radiating structure whose electrical length introduces frequency-selective coupling [23]. In simplified analyses, this behavior is often captured by a quarter-wave resonance at wavelengths comparable to the cable length, but in practice, the effective coupling response is shaped by additional parasitic contributions beyond the cable alone. For the purposes of our attack model, we therefore treat the cable coupling as a bandpass-like function C(f) whose passband depends on the installation geometry, without committing to a specific analytical form; Section 6.5 characterizes this dependence experimentally.

Combining the cable coupling response C(f) and the ESC input-path response H(f), the effective disturbance amplitude at the thresholding node can be expressed as

(7) $${\left|n_{k}\right|}_{\mathrm{eff}}\approx \left|C(f_{\mathrm{inj}})\cdot H(f_{\mathrm{inj}})\right|\cdot A_{\mathrm{inj}}.$$

The product |C(f)·H(f)| is the end-to-end transfer function from the injection source to the thresholding node. Its maxima correspond to frequencies at which a given injection amplitude produces the largest disturbance, and consequently the largest timing shift according to (4).

### 5.3. Existence of an Optimal Attack Frequency and Minimum Amplitude

The preceding two subsections imply a concrete attack condition, but also a concrete tension for the attacker. On one hand, the timing shift in Equation (4) grows with the disturbance amplitude at the PWM edge; for a sinusoidal injection, edges are more effectively corrupted when the injection period is short compared to the edge transition time, favoring higher injection frequencies. On the other hand, the ESC’s input-path response H(f) attenuates high-frequency components above its cutoff, so a disturbance at an arbitrarily high frequency does not reach the thresholding node. The attacker therefore faces a trade-off: edge corruption pushes toward higher frequencies, while input-path filtering pushes toward lower frequencies. The optimal attack frequency lies where these two effects balance, which coincides with the peak of the cascade response |C(f)·H(f)| and therefore the minimum of $A_{\mathrm{inj}}^{★}(f)$.

Inducing motor stoppage through any of the failure paths described in Section 5.1 requires the cumulative timing error $\left|e_{k}\right|$ to exceed a device-specific threshold $e_{\mathrm{stop}}$, which in turn requires a sufficiently large effective disturbance at the thresholding node. Combining (4) with (7) and requiring $|e_{k}|\geq e_{\mathrm{stop}}$, the minimum injection amplitude at frequency $f_{\mathrm{inj}}$ that induces motor stoppage satisfies

(8) $$A_{\mathrm{inj}}^{★}(f_{\mathrm{inj}})\;\propto \;\frac{1}{\left|C(f_{\mathrm{inj}})\cdot H(f_{\mathrm{inj}})\right|},$$

where the proportionality factor absorbs the edge slope, the logic-level swing, and $e_{\mathrm{stop}}$, all of which are device-specific but approximately constant within the susceptible regime. Equation (8) predicts that the minimum attack amplitude curve, plotted as a function of $f_{\mathrm{inj}}$, follows the reciprocal of the end-to-end transfer function from source to thresholding node.

We emphasize that (8) is intended as a mechanistic, descriptive relation rather than a self-contained quantitative predictor. The product |C(f)·H(f)| is not fitted to two free functions whose factors are then inferred from the same susceptibility data; instead, the two stages are constrained by independent evidence. The cable coupling response C(f) is characterized directly through the induced-voltage measurement of Section 6.6, in which the disturbance voltage developed on the PWM cable under radiated injection is measured as a function of frequency and standoff distance, independently of any motor-stoppage observation. The input-path stage H(f) is constrained by the conducted-injection sweep of Section 6.2, which confines the injected energy to the PWM conductor and therefore reflects an effective input-path response dominated by H(f). Used together, these measurements allow the cascade model to be compared against data rather than tuned to reproduce it.

This prediction has two practical implications that frame the experimental evaluation. First, for each ESC, there exists a specific frequency $f_{\mathrm{inj}}^{★}$ at which |C·H| is maximized and the required injection amplitude is minimized; this frequency characterizes the optimal attack point against that device, and its location is determined jointly by the cable length and the ESC’s input-path design. Second, the shape of $A_{\mathrm{inj}}^{★}(f)$ across frequency reflects the combined effects of the cable coupling and the ESC’s input-path filtering, so comparing this shape across different ESCs reveals how their design attributes translate into measurable differences in attack threshold. Section 6 presents these comparisons for multiple commercial ESCs.

## 6. EM Injection Susceptibility of BLDC-ESCs

This section experimentally characterizes the attack conditions under which electromagnetic injection induces motor stoppage across a representative set of commercial BLDC-ESCs. The experiments address four questions that directly reflect the theoretical predictions of Section 5: (i) does each ESC exhibit a characteristic pairing of susceptible frequency and minimum injection amplitude, as predicted by the cascade transfer function in Equation (8); (ii) do these $\left(f,\;A^{★}\right)$ pairs differ systematically across ESCs in a manner consistent with their design; (iii) does radiated injection at the frequencies identified under conducted injection reproduce motor stoppage, as required for a non-invasive deployment-phase attack consistent with the attacker model in Section 4; and (iv) how do deployment-level factors such as the PWM cable length shape the susceptible frequencies.

### 6.1. Experimental Setup and Procedure

**Figure 3:**
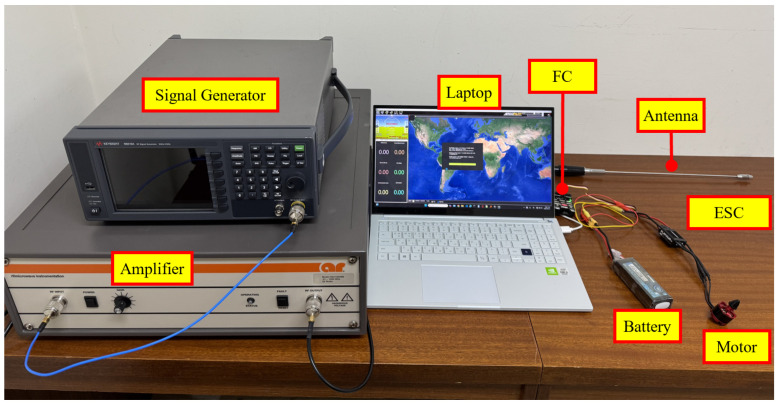
Experimental setup for conducted and radiated EMI injection against the FC-to-ESC PWM command path.

The experimental setup is shown in Figure 3, and the instruments used are summarized in Table 1. The victim system consists of a Pixhawk-class FC [24] connected to the ESC under test via a PWM signal cable, with the ESC driving a BLDC motor powered from a lithium-polymer (LiPo) battery. The FC outputs a constant mid-throttle PWM command (50% throttle) so that the motor operates at a nominal steady-state speed in the absence of interference, and motor state is monitored in real time through the Mission Planner ground control software [25]. Motor stoppage is identified as the condition in which the motor ceases to rotate, i.e., its rotational speed collapses from this 50%-throttle nominal speed to zero under injection.

The attack chain comprises a narrowband RF signal generator feeding a broadband RF power amplifier, whose output is routed either (i) directly onto the PWM signal cable between the FC and the ESC, for conducted (wired) injection that isolates the PWM interpretation path from free-space coupling, or (ii) to a wideband portable antenna (Komunica HF-PRO-1, 0 dBi) for radiated (wireless) injection reflecting a realistic non-invasive attack. For the radiated experiments, the antenna was mounted with its radiating element parallel to, and co-planar with, the FC-to-ESC cable run (i.e., matched linear polarization), at a nominal standoff of 0.5 m between the antenna feed point and the cable unless otherwise stated. Because the free-space wavelength in the low band (10–35 MHz, *λ* ≈ 8.6–30 m) is far larger than this standoff, the radiated experiments operate in the reactive/radiating near-field rather than the far-field; the induced-voltage characterization of Section 6.6 quantifies the resulting coupling as a function of distance and frequency.

Five commercial BLDC-ESCs, summarized in Table 2, are evaluated. The selection deliberately spans a wide range of application classes—from a low-cost consumer toy drone ESC (GT Drone EC-X3) to high-end helicopter and airplane ESCs (Castle Phoenix Edge 75, Hobbywing Platinum 60A V4)—to expose how differences in PWM input-path design translate into differences in attack conditions. All ESCs use analog PWM throttle decoding.

The experiments follow a two-stage procedure that mirrors the two-phase attacker model of Section 4 and the path-isolation strategy of Section 4. In Stage 1 (Section 6.2), we perform conducted injection on each ESC and sweep the injection frequency to identify the device-specific susceptible frequency $f^{★}$ at which the minimum stoppage amplitude $A_{\min}^{★}$ is observed. Because conducted injection confines the injected energy to the PWM conductor and its ground return, any motor anomaly in Stage 1 is attributable to corruption of the PWM interpretation path itself rather than to interference with power wiring, motor phases, or sensor-FC buses; this stage therefore characterizes an effective input-path response dominated by H(f)—up to contributions from the injection fixture, ground return, and common-mode coupling that conducted injection does not fully eliminate—for each ESC, up to a geometry-independent proportionality. This stage corresponds to the offline characterization phase of the attacker model, in which the attacker profiles a representative target device in a controlled setting.

In Stage 2 (Section 6.3 and Section 6.4), we apply radiated injection and sweep the frequency around the $f^{★}$ identified in Stage 1, recording whether and at what amplitude motor stoppage is observed under non-invasive conditions. This stage corresponds to the deployment phase, in which the attacker acts on an operational UAV without physical contact, and the resulting thresholds reflect the full cascade |C(f)·H(f)| from source to thresholding node. Comparison of Stage 1 and Stage 2 results thus attributes the difference primarily to the cable coupling contribution C(f), recognizing that conducted injection itself retains residual fixture, ground-return, and common-mode contributions and so does not yield H(f) in perfect isolation. Section 6.5 then varies the PWM cable length directly to confirm that the cable coupling response shifts with the installation geometry.

Unless otherwise noted, the PWM signal cable between the FC and the ESC has a length of 45 cm, consisting of unshielded servo-grade three-conductor wiring routed in free space. This length is used for all Stage 1 conducted-injection experiments (Section 6.2) and for the Stage 2 radiated-injection experiments in Section 6.3 and Section 6.4. The cable-length dependence of the susceptible frequency is examined separately in Section 6.5, where the cable length is varied from 45 cm to 125 cm.

Throughout, the reported injection amplitude $A_{\mathrm{inj}}$ (and the threshold $A_{\min}^{★}$) refers to the amplitude at the output of the RF chain feeding the injection point—i.e., the conducted drive level applied to the PWM cable in Stage 1 and the amplifier output delivered to the transmitting antenna in Stage 2—expressed as the peak voltage of the sinusoidal source. It is thus a source-referenced quantity and not the voltage developed at the ESC thresholding node, which is the source attenuated by the cascade |C(f)·H(f)|; the disturbance voltage actually induced on the cable is measured separately in Section 6.6. Each reported minimum amplitude $A_{\min}^{★}$ is the average of five repeated measurements taken at the same operating point: at each frequency, the injection amplitude is raised in discrete steps until motor stoppage occurs, this measurement is repeated five times, and the reported threshold is the mean of the five trials. Reporting the averaged value, rather than a single-shot reading, ensures that the tabulated thresholds reflect repeated trials and are robust to trial-to-trial variation.

### 6.2. Stage 1: Susceptible Frequency Search via Conducted Injection

**Figure 4:**
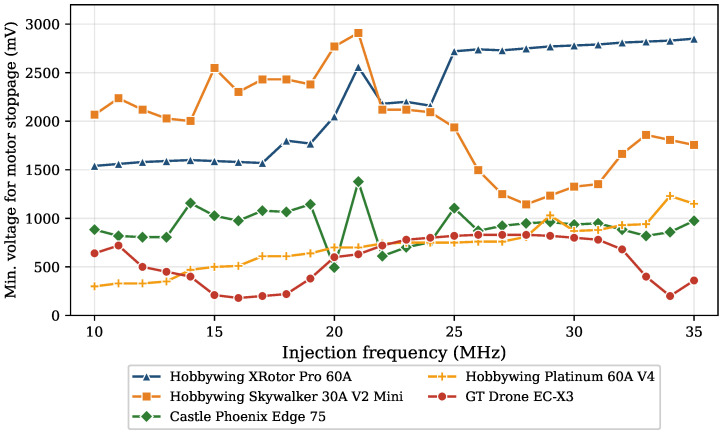
Stage 1 conducted-injection sweep over the low-frequency band (10–35 MHz) for five commercial ESCs. Values indicate the minimum injection amplitude inducing motor stoppage at each frequency.

Figure 4 shows the Stage 1 results over the low-frequency band (10–35 MHz). All five ESCs exhibit motor stoppage somewhere within this band under conducted injection, with device-specific $A_{\min}^{★}$ ranging from 180 mV (GT Drone EC-X3 at 16 MHz) to 1540 mV (XRotor Pro 60A at 10 MHz)—a factor of roughly nine between the most and least susceptible units. The shapes of the individual curves differ substantially. EC-X3 exhibits a pronounced dip near 16 MHz with thresholds below 1 V across most of the band. Platinum 60A V4 exhibits its lowest threshold at 10 MHz (300 mV) and a slow monotonic rise with frequency. Edge 75 shows a sharp local minimum at 20 MHz (494 mV) surrounded by higher thresholds above 800 mV. Skywalker 30A V2 Mini’s most sensitive region within this band lies near 28–29 MHz (1144 mV minimum), while its threshold remains above 1.9 V elsewhere. Among the five ESCs, however, its low-band susceptibility is only moderate, with a minimum threshold well above those of EC-X3, Platinum 60A V4, and Edge 75. XRotor Pro 60A requires the largest amplitude overall, with its minimum at the low edge of the band. Each curve is consistent with a device-specific H(f) response whose shape is determined by the ESC’s input-path filter, parasitic reactances, and internal logic thresholds; no two ESCs exhibit the same spectral susceptibility.

**Figure 5:**
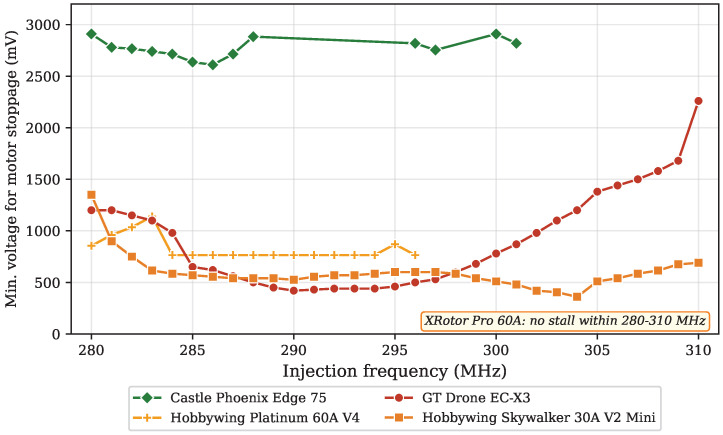
Stage 1 conducted-injection sweep over the high-frequency band (280–310 MHz). XRotor Pro 60A showed no motor stoppage in this band up to 3 V and is omitted from the plot.

Figure 5 shows the corresponding Stage 1 results over the high-frequency band (280–310 MHz). Four of the five ESCs exhibit motor stoppage in this band: EC-X3 (minimum 420 mV at 290 MHz), Skywalker (360 mV at 304 MHz), Platinum (765 mV at 284 MHz), and Edge 75 (2610 mV at 286 MHz). Only XRotor Pro 60A remained immune throughout 280–310 MHz at injection amplitudes up to 3 V. Notably, Skywalker is the most susceptible of the five ESCs in the high band, with a threshold below that of EC-X3, despite being one of the less susceptible ESCs in the low band. This reordering is a direct indication that H(f) does not admit a single scalar susceptibility score per ESC: each ESC has its own spectral shape, with distinct susceptibility peaks at low and high frequencies that do not necessarily coincide.

Table 2 summarizes the Stage 1 results. These per-ESC $\left(f^{★},\;A_{\min}^{★}\right)$ pairs serve as the reference points for Stage 2: each ESC is attacked in Stage 2 at its own Stage 1 low-band $f^{★}$ to test whether the same vulnerability remains exploitable under radiated injection.

**Table 2:** Stage 1 conducted-injection sweep results. For each ESC, we report the susceptible frequency $f^{★}$ and corresponding minimum injection amplitude $A_{\min}^{★}$ in each of the two measurement bands. Entries marked “—” indicate no motor stoppage was observed up to the 3 V maximum injection amplitude.

Manufacturer	Model	Low Band (10–35 MHz)		High Band (280–310 MHz)	
		$f^{★}$ (MHz)	$A_{\min}^{★}$ (mV)	$f^{★}$ (MHz)	$A_{\min}^{★}$ (mV)
GT Drone	EC-X3	16	180	290	420
Hobbywing	Skywalker 30A V2 Mini	28	1144	304	360
	XRotor Pro 60A	10	1540	—	—
	Platinum 60A V4	10	300	284	765
Castle Creations	Phoenix Edge 75	20	494	286	2610

### 6.3. Stage 2: Radiated Attack Validation

**Figure 6:**
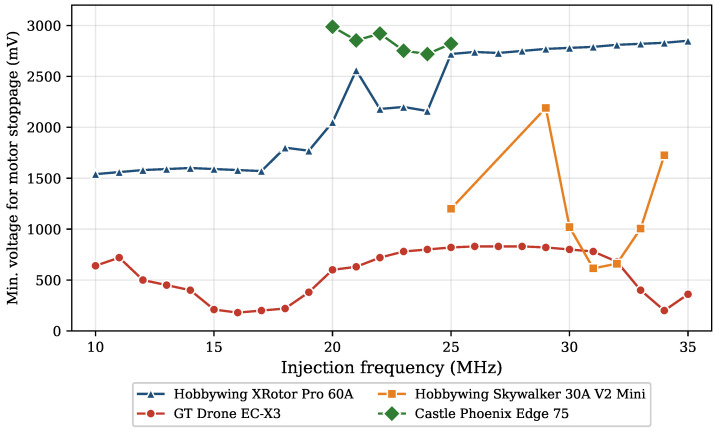
Stage 2 radiated-injection sweep over the low-frequency band (10–35 MHz) for four of the five commercial ESCs. Hobbywing Platinum 60A V4 is omitted from this plot because it showed no motor stoppage within 10–58 MHz at injection amplitudes up to 3 V; its susceptible frequency under radiated injection lies at 59–60 MHz with $A_{\min}^{★}=1395$ mV (see Table 3).

Figure 6 shows the Stage 2 results in the low-frequency band, obtained by placing the wideband antenna at the standoff and polarization described in Section 6.1 (nominally 0.5 m, co-planar matched polarization) and sweeping the injection frequency and amplitude. Three qualitative differences distinguish Stage 2 from Stage 1. First, the radiated $A^{★}$ values are generally larger than their conducted counterparts at corresponding frequencies. Second, the bands over which each ESC remains susceptible are generally narrower: Edge 75, for instance, narrows from a broad conducted sensitivity to a 20–25 MHz window under radiation. Third, and most consequential, Platinum 60A V4 exhibits no motor stoppage anywhere within 10–58 MHz at injection amplitudes up to 3 V, despite its low conducted threshold of 300 mV at 10 MHz; stoppage under radiated injection is observed only when the sweep is extended to 59–60 MHz, where $A_{\min}^{★}=1395$ mV. For clarity, Platinum 60A V4 is therefore omitted from the 10–35 MHz plot in Figure 6, and its susceptible point is reported in Table 3 and discussed separately in the text.

Table 3 compares the Stage 1 and Stage 2 low-band results. These differences are not noise but a structural consequence of the cascade model. Under conducted injection, the effective disturbance at the thresholding node is shaped by H(f) alone, whereas under radiated injection it is shaped by the full cascade |C(f)·H(f)|. The cable coupling response C(f) is bandpass-like with its passband determined by the geometry of the PWM cable and its ground return, so radiated injection effectively filters the attacker’s source through this additional stage.

Two observations illustrate this interpretation particularly clearly. The first is apparent ESC immunity created by C(f). Platinum 60A V4’s conducted susceptibility is strongest at 10 MHz, where the free-space wavelength is approximately 30 m and the FC-to-ESC cable (of order $10^{-1}$ m) is electrically very short; the radiated coupling into the cable at this frequency is therefore weak, and the intrinsic input-path susceptibility of Platinum is effectively hidden under radiated testing. The susceptible frequency shifts from 10 MHz (conducted) to 59–60 MHz (radiated), corresponding to a wavelength at which the cable length is a more efficient fraction of a quarter wave. The second is apparent ESC susceptibility amplified by C(f). For Skywalker 30A V2 Mini, the radiated threshold of 615 mV at 31 MHz is lower than the conducted minimum of 1144 mV at 28 MHz. This inversion indicates that at 31 MHz the cable operates near a standing-wave resonance that amplifies the incident field at the ESC input relative to the source, more than compensating for the radiation-to-wire coupling loss. Both observations confirm that under the attacker model, observed susceptibility is a property of the ESC–cable pair at a specific frequency, not of the ESC in isolation.

Despite these differences, the susceptible frequencies identified in Stage 1 remain useful predictors of Stage 2 vulnerability for the majority of ESCs: EC-X3 and XRotor show near-identical $f^{★}$ under the two injection modes, and the Stage 2 $f^{★}$ values for Skywalker and Edge 75 sit within 3 MHz of their Stage 1 counterparts. This partial agreement supports the path-isolation argument of Section 4: for most ESCs in the configuration tested, the results are consistent with the PWM interpretation path being the primary entry point in both conducted and radiated cases, rather than some alternative coupling path that activates only under radiated conditions.

### 6.4. Cross-Device Comparison

**Figure 7:**
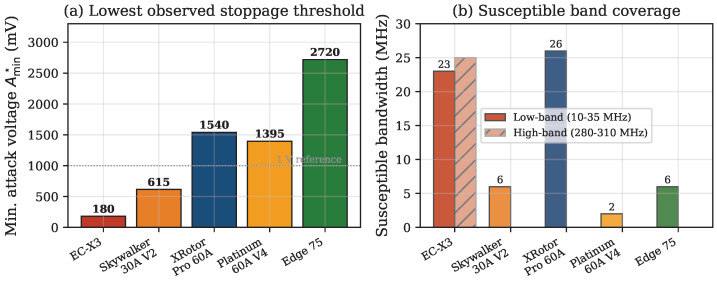
Cross-device comparison of attack thresholds and susceptible bandwidths under radiated injection.

Figure 7 summarizes the Stage 2 radiated results across the five ESCs along two dimensions: the lowest observed stoppage amplitude in Figure 7a, and the contiguous susceptible bandwidth in Figure 7b. The radiated $A_{\min}^{★}$ values span approximately a factor of fifteen across the devices, from 180 mV (EC-X3) to 2720 mV (Edge 75). The susceptible bandwidth in the low-frequency band ranges from approximately 2 MHz for Platinum 60A V4—which is effectively out-of-band within 10–35 MHz and exhibits a narrow 59–60 MHz window just above it—to more than 20 MHz for EC-X3.

This spread is substantial, but it is not a simple monotonic function of ESC application class or market price. Stage 1 establishes that the per-ESC H(f) shapes reorder between the low and high bands: Platinum moves from second-most susceptible in the low band to third in the high band, and Skywalker moves from second-most robust in the low band to most susceptible in the high band. Stage 2 further reorders the devices by combining H(f) with the device-specific cable coupling realized in the deployment. The resulting Stage 2 ranking, therefore, reflects the joint design of ESC, cable, and injection frequency, not a single scalar “robustness” score per ESC.

Two more specific observations follow from this picture. First, Edge 75 exhibits a narrow radiated susceptible band in the low-frequency region (20–25 MHz) with a relatively high minimum threshold of 2720 mV at 23 MHz. Its conducted threshold at 20 MHz, however, is only 494 mV—by no means the highest among the five ESCs—which indicates that its intrinsic input path is not exceptionally robust. The apparent robustness under radiated injection is therefore better attributed to a geometric mismatch between the cable coupling response C(f) and the ESC’s intrinsic susceptible frequency near 20 MHz, rather than to a particularly steep H(f) roll-off. Second, Platinum 60A V4, which Hobbywing positions as a high-end unit with improved noise immunity through an independent DC regulator, does indeed exhibit the most restricted radiated susceptibility; but Stage 1 reveals that its intrinsic input path is broadly susceptible at low frequency, so its radiated robustness derives primarily from the geometric mismatch between that susceptible range and the radiated coupling efficiency rather than from input filtering alone. In short, the observed wireless attack threshold is not an inherent property of the ESC; it is the composition of the ESC’s H(f) with the installation’s C(f), and either factor can dominate the effective susceptibility.

### 6.5. Effect of PWM Cable Length on Susceptible Frequency

The preceding Stage 2 results establish that the cable coupling response C(f) is a significant contributor to observed radiated susceptibility, and that its mismatch with a given ESC’s H(f) can alternately hide or amplify intrinsic vulnerabilities. This subsection tests the C(f) component directly by varying the PWM cable length while keeping the ESC fixed. We perform radiated injection on the GT Drone EC-X3—chosen for its broad low-band susceptibility that makes frequency migration easy to observe—across five PWM cable lengths (45, 65, 85, 105, and 125 cm) over the 10–35 MHz band.

**Figure 8:**
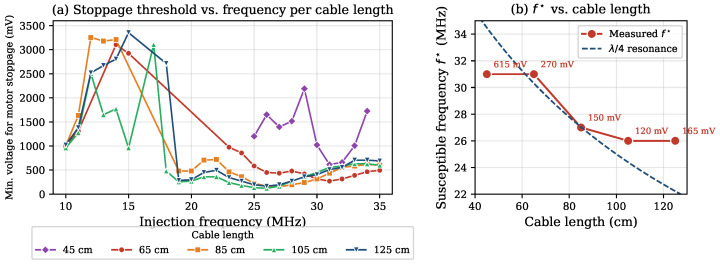
Effect of PWM cable length on radiated-injection susceptibility of the GT Drone EC-X3. (**a**) Minimum injection amplitude $A_{\min}^{★}$ for motor stoppage as a function of frequency for five cable lengths. (**b**) Device-specific susceptible frequency $f^{★}$ versus cable length, with a descriptive transmission-line fit based on a loaded quarter-wave model that includes an empirical extra electrical length $L_{\mathrm{extra}}\approx 1$ m (see text in Section 6.5).

Figure 8a shows three coupled trends. First, $f^{★}$ shifts downward as the cable lengthens: 31 MHz at 45 and 65 cm, 27 MHz at 85 cm, and 26 MHz at 105 and 125 cm. Second, $A_{\min}^{★}$ decreases from 615 mV at 45 cm to 120 mV at 105 cm—a fivefold reduction—before rising slightly to 165 mV at 125 cm. Third, the susceptible bandwidth expands from 10 of the 26 tested frequencies at 45 cm to 25 of 26 at 105 cm.

These trends are qualitatively consistent with a transmission-line interpretation of C(f): longer cables should correspond to lower resonant frequencies, and the data reproduce the expected downward shift of $f^{★}$. We stress, however, that the cable is *not* well modeled as an isolated quarter-wave resonator, and we do not claim quantitative agreement with such a model. A pure λ/4 prediction using a propagation velocity $v_{\mathrm{eff}}\approx 2\times 10^{8}$ m/s places the resonance for a 45–125 cm cable in the 40–110 MHz range, well above the 26–31 MHz we observe—a discrepancy too large to be reconciled by the cable conductor alone. The cable is therefore better understood as one element of a *loaded radiating structure* whose effective electrical length is set jointly by the conductor, the connectors, the chassis coupling, and the loading impedance of the ESC input. The descriptive fit in Figure 8b makes this explicit: matching the data requires an effective extra electrical length $L_{\mathrm{extra}}\approx 1$ m, which is comparable to or larger than the physical cable itself. Rather than treating this as a calibrated cable resonance, we read the large $L_{\mathrm{extra}}$ as direct evidence that the connector, chassis, and ESC-input contributions—not the conductor length—dominate the coupling structure. Accordingly, we use the model only to capture the *direction* of the $f^{★}$ shift with cable length (longer cable → lower $f^{★}$), which the data confirm, and not to predict $f^{★}$ quantitatively. The decrease of $A_{\min}^{★}$ with cable length reflects the cable’s role as a receiving antenna under radiated injection, which couples incoming electromagnetic energy more efficiently near its resonance; the slight rise at 125 cm indicates that the 105 cm configuration lies closest to the peak of the coupling response within the 10–35 MHz window, and that longer cables begin to detune. The broadening of the susceptible band with cable length is consistent with longer cables supporting higher-order modes in addition to the primary quarter-wave, producing standing-wave amplification across a wider set of frequencies at the ESC input.

Taken together with the Stage 2 cross-device results, this experiment confirms qualitatively that the same ESC presents different radiated susceptibilities when installed with different cable geometries. While the precise spectral shape of C(f) remains beyond what our black-box measurements can resolve, the direction and magnitude of the observed $f^{★}$ shift are consistent with the cascade model developed in Section 5 and with the design-attribute discussion of Section 6.4.

### 6.6. Direct Measurement of Cable Coupling and Attack Standoff

The Stage 1 and Stage 2 experiments infer the role of C(f) indirectly, by comparing stoppage thresholds across injection modes. To address the concern that the cascade model could otherwise be satisfied by an arbitrary factorization of C(f) and H(f), we additionally measure the disturbance voltage that the radiated injection actually develops on the PWM cable. With the victim system held at the radiated configuration of Section 6.1 and the cable length fixed at 50 cm, we probe the voltage induced on the PWM conductor and record its amplitude while sweeping the injection frequency across both bands and varying the antenna standoff distance from 0.5 m to 2 m. This measurement reflects the source-to-cable coupling stage C(f) directly, without reference to whether a motor stoppage occurs, and therefore provides an independent constraint on the cascade model rather than a quantity fitted to the susceptibility data.

**Figure 9:**
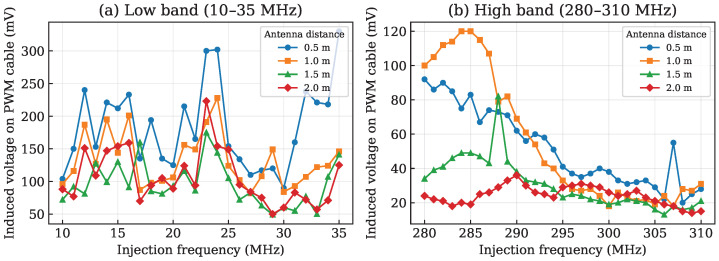
Directly measured disturbance voltage induced on the PWM cable under radiated injection, as a function of injection frequency and antenna standoff distance (0.5–2 m). (**a**) Low band (10–35 MHz); (**b**) high band (280–310 MHz). The PWM cable length is fixed at 50 cm. These curves characterize the cable coupling response C(f) and its distance dependence independently of any motor-stoppage observation.

Figure 9 shows the results. Two features are relevant to the attack model. First, the induced voltage is strongly frequency-selective: in the low band it ranges from roughly 50 to 330 mV with pronounced structure—at 0.5 m it peaks near 23–24 MHz and again toward 35 MHz—whereas in the high band it is markedly smaller, on the order of 15–120 mV, and decreases with frequency across most of the 280–310 MHz range. This confirms experimentally that the PWM cable acts as a bandpass-like coupling element, favoring the low band, consistent with the C(f) component posited in Section 5.2: the several-fold larger coupling in the low band is the same effect that makes the low-band susceptible frequencies more readily exploitable under radiated injection. Second, the induced voltage decreases with antenna standoff: the low-band, frequency-averaged induced voltage falls from approximately 184 mV at 0.5 m to about 106 mV at 2 m, with the decay being neither monotone at every frequency nor following a simple inverse-distance law—as expected for near-field coupling, where a standing-wave structure on the cable and reactive field components dominate over a clean far-field roll-off.

These measurements also support a first-order standoff estimate for the radiated attack. At EC-X3’s low-band susceptible frequency of 16 MHz, the induced voltage at 0.5 m reaches 233 mV, which already exceeds the 180 mV conducted stoppage threshold of this device; at 2 m it remains at 159 mV, of the same order as the threshold. For the most susceptible devices, therefore, the present antenna and source produce a stop-equivalent disturbance at the cable at standoff distances on the order of 1–2 m. Because induced voltage scales with the radiated field, and field strength in turn scales with the square root of effective radiated power, increasing the attacker’s effective radiated power by a factor of *k* raises this disturbance by approximately $\sqrt{k}$, extending the standoff at which the same cable voltage is reached. We caution that these figures apply to the unshielded, free-space cable routing used here and represent a favorable rather than worst-case geometry; nonetheless, they bound the order of magnitude of the near-field standoff and confirm that the radiated threat is not confined to direct contact with the wiring.

## 7. Discussion

This section reflects on the implications of our findings and discusses the limitations of the current study.

### 7.1. Implications for UAV Propulsion Security

Our results demonstrate that the PWM command path between the FC and the ESC is a tractable attack surface at modest injection power, provided the attacker can identify the device’s susceptible frequency. The conducted-injection experiments establish that motor stoppage is achievable with sub-volt disturbance amplitudes on specific ESCs, and the radiated-injection experiments confirm that the same vulnerability remains exploitable in a genuinely non-invasive deployment. Because the PWM interface is widely used in commodity and legacy-compatible multirotor platforms, this attack surface is not confined to a particular manufacturer or product line but is a structural property of PWM-based propulsion.

The cross-device comparison further shows that susceptibility varies substantially—by more than an order of magnitude in minimum injection amplitude—depending on the ESC’s input-path design and the cable configuration. Comparing Stage 1 and Stage 2, however, reveals that the attacker-observable radiated threshold is not a direct measure of an ESC’s intrinsic susceptibility: Platinum 60A V4 appears nearly immune below 58 MHz under radiated injection, yet is broadly susceptible under conducted injection, with motor stoppage achievable at 300 mV near 10 MHz. This gap is attributable to the cable coupling response C(f), which can alternately hide or amplify intrinsic vulnerabilities depending on the geometric match between the cable and the injection frequency. A single susceptibility measurement, therefore, cannot be generalized across UAV models, but the underlying factors (input RC filter, PWM cable length, logic-level swing) are observable and therefore enumerable. An adversary who can survey target hardware in an offline characterization phase can plausibly build a lookup table of optimal attack parameters for commercially available platforms.

### 7.2. Mitigation Strategies

The timing-error model and the cascade of cable and input-path transfer functions jointly suggest several mitigation directions, organized by the layer at which they intervene. Because the effective disturbance at the thresholding node is the product $\left|C\left(f\right)\cdot H\left(f\right)\right|\cdot A_{\mathrm{inj}}$, attenuating either stage raises the amplitude—or reduces the standoff—an attacker requires.

#### 7.2.1. Signal Integrity at the PWM Line

Reducing the coupling efficiency C(f) at the exploited frequencies attacks the cascade at its first stage. Shielding the PWM cable, routing the signal as a twisted differential pair with a defined ground return, and adding ferrite clamps all suppress common-mode pickup on the conductor [23]; differential signaling in particular rejects the common-mode disturbance that threshold-based decoding is sensitive to. Because our experiments show that cable length directly shifts the resonant peaks of C(f), deliberate cable-routing choices—shortening the FC-to-ESC run where mechanical constraints allow, or avoiding lengths whose resonances fall in bands an attacker can readily generate—move the coupling passband away from the most convenient attack frequencies.

#### 7.2.2. ESC Input-Path Hardening

At the second stage, an input RC low-pass filter with a cutoff well below the observed susceptible bands reduces the effective disturbance reaching the thresholding node, at the cost of slightly slower edge response. Adding input hysteresis (a Schmitt-trigger front-end with a wider threshold separation) further suppresses the multiple-crossing and small-shift failure modes of Section 5.1, since a disturbance must then exceed the hysteresis band to alter the decoded edge. Our Stage 1 results show that conducted thresholds span roughly a factor of nine across the ESCs tested, confirming that input-path design is a meaningful axis for hardening. The Stage 1/Stage 2 comparison, however, shows that apparent wireless immunity can arise from a mismatch between C(f) and the ESC’s susceptible frequency rather than from strong input filtering alone: a device that appears robust under one installation may be vulnerable under a more favorable attack geometry. Input-path hardening is therefore most effective when combined with cable-side mitigation, so that both stages of the cascade attenuate the attacker’s signal. More actively, inserting a digital isolator or regenerating buffer between the coupling zone and the ESC’s MCU removes analog perturbation accumulated on the cable, since the downstream logic sees only a reconstructed digital signal.

#### 7.2.3. Protocol-Level Defense

The deepest mitigation is to replace threshold-timing-based PWM decoding with a digital protocol that includes framing and integrity checks, such as DShot [26]. While this is a longer-term solution requiring coordinated changes in FCs and ESCs, our results add a concrete security argument to the existing performance rationale for digital ESC protocols.

None of these mitigations is individually sufficient against a determined attacker, but their combination meaningfully raises the cost—in injection amplitude, proximity, or required hardware capability—of an effective attack.

### 7.3. Limitations and Future Work

Several aspects of our analysis deserve further investigation.

First, the experiments characterize the attack outcome at the level of observable motor behavior, specifically motor stoppage, monitored through the flight-controller telemetry and ground-control software rather than through a dedicated motor-speed transducer. We have not instrumented the internal state of the ESC to determine which of the three failure paths identified in Section 5.1—invalid-signal failsafe, minimum-throttle decoding, or edge-capture timeout—is triggered in each case, nor have we recorded the decoded PWM pulse width directly to show its corruption under interference. Correlating each observed stoppage with a specific mechanism, and quantifying the rotational-speed collapse itself, would require capturing the thresholding-node waveform at the ESC input together with a synchronized motor-speed trace (e.g., from an optical tachometer or ESC telemetry) under representative injection conditions. Such a synchronized pulse-width-and-speed measurement would simultaneously (i) demonstrate PWM pulse-width corruption directly, (ii) tie each stoppage to its specific failure path, and (iii) replace the qualitative “stoppage” label with a quantitative speed measurement; it is a direct and concrete extension of the present black-box characterization and is left for future work.

Second, the timing-error model is a first-order approximation valid in a weak-coupling regime. At high injection amplitudes, nonlinear effects such as Schmitt-trigger hysteresis, waveform clipping, and multiple threshold crossings near PWM edges may dominate and produce qualitatively different stop transitions. A systematic characterization of these nonlinear regimes, including abrupt onset behaviors, is left for future work.

Third, our cable-length study examines five lengths in the 45–125 cm range on a single ESC model. A broader sweep across cable types (with different dielectric properties and therefore different propagation velocities), shielding configurations, and routing geometries would more fully characterize the C(f) component of the cascade model. In addition, our model captures only the qualitative direction of the $f^{★}$ shift with cable length: a full quantitative prediction of $f^{★}$ from cable geometry, including the effective electrical contributions of connectors, chassis, and ESC input loading, requires a distributed electromagnetic analysis that is beyond the scope of the present work. We also note that the cable lengths examined here (45–125 cm) are longer than the 5–30 cm FC-to-ESC lengths typical of compact multirotors, chosen to place the resonant frequencies within the operating range of our antenna; characterizing C(f) for shorter, deployment-realistic cable lengths is a natural direction for future work.

## 8. Conclusions

This paper investigated electromagnetic interference as a physical-layer attack surface against UAV propulsion, in which a sinusoidal disturbance on the FC-to-ESC PWM line can corrupt the ESC’s pulse-width interpretation and induce motor stoppage. Whether such an attack succeeds depends jointly on the injection frequency, the injection amplitude, and the particular ESC and cable configuration targeted; the same source conditions that halt one ESC leave another unaffected, and a given ESC that appears robust under one measurement setup may be vulnerable under another. To make this dependence explicit, we developed a timing-error model whose key prediction is a device-specific minimum-amplitude curve shaped by two frequency-selective components: the ESC’s input-path response H(f) and the PWM cable’s coupling response C(f).

We evaluated this framework on five commercial ESCs through a two-stage experimental strategy. In Stage 1, conducted injection largely removes the cable coupling and probes an effective input-path response dominated by H(f), revealing that each ESC has its own susceptibility curve and that attack thresholds vary by more than an order of magnitude across devices. In Stage 2, radiated injection reintroduces the cable, and the resulting differences from Stage 1 are attributed primarily to C(f)—showing cases where the cable masks an ESC’s susceptibility and cases where it amplifies that susceptibility. A separate cable-length experiment confirms that C(f) shifts with the installation geometry in qualitative agreement with a transmission-line resonance interpretation, even though the precise spectral shape requires additional contributions beyond the cable alone—including connector, chassis, and ESC input-loading effects—to match the data quantitatively. Together, these results demonstrate that the attacker-observable threshold is not a property of the ESC in isolation but of the ESC–cable system at a chosen frequency, determined jointly by the ESC’s input-path response and the cable’s coupling response rather than by any single intrinsic property. We note that the cable lengths studied here (45–125 cm) exceed the 5–30 cm typical of compact multirotors; this range was dictated by our antenna’s operating band, and extending the characterization of C(f) to shorter, deployment-realistic cables—where resonances would move to higher frequencies that demand different attack hardware—is a priority for future work.

## Figures and Tables

**Table 1:** Equipment used in the experimental setup.

Role	Model	Key Specification
Flight controller	Pixhawk-class	PX4/ArduPilot-compatible
Ground control station	Mission Planner	PWM command monitoring
Motor	BLDC	driven by ESC under test
Battery	3S LiPo	11.1 V nominal, 2200 mAh
Radio-frequency (RF) signal generator	Keysight N9318A	narrowband sinusoidal source
RF power amplifier	AR 50U1000	1–1000 MHz, 50 W
Antenna (radiated)	Komunica HF-PRO-1	7–430 MHz, 0 dBi, 50 Ω

**Table 3:** Per-ESC comparison of Stage 1 (conducted/wired) and Stage 2 (radiated/wireless) low-band results.

Manufacturer	Model	Stage 1 (Conducted)		Stage 2 (Radiated)	
		$f^{★}$ (MHz)	$A_{\min}^{★}$ (mV)	$f^{★}$ (MHz)	$A_{\min}^{★}$ (mV)
GT Drone	EC-X3	16	180	16	180
Hobbywing	Skywalker 30A V2 Mini	28	1144	31	615
	XRotor Pro 60A	10	1540	10	1540
	Platinum 60A V4	10	300	60	1395
Castle Creations	Phoenix Edge 75	20	494	23	2720

## Data Availability

The data presented in this study are available on request from the authors.
